# The Relationship between Marital Satisfaction and Spiritual Well-Being of Chinese Older Adults: The Mediating Effect of Psychological Security and Aging Expectations

**DOI:** 10.3390/bs14100949

**Published:** 2024-10-15

**Authors:** Longxing Tang, Yachi Yang, Zhiwei An, Yalian Huang, Ping Tang

**Affiliations:** 1 School of Psychology, Chengdu Medical College, Chengdu 610500, China; tanglongxing@cmc.edu.cn (L.T.); yangyachi@cmc.edu.cn (Y.Y.); azw15764307177@163.com (Z.A.); 2 Sichuan Applied Psychology Research Center, Chengdu Medical College, Chengdu 610500, China

**Keywords:** older adults, marital satisfaction, spiritual well-being, psychological security, aging expectations

## Abstract

This study explores the relationship between marital satisfaction and spiritual well-being in older adults and the role of psychological security and aging expectations in older adult relationships. A convenient sample cross-sectional research method collected data from 367 older adults in Sichuan Province, China. A chain mediation model was constructed using quantitative analysis methods to test the relationship between marital satisfaction and older adults’ spiritual well-being and the potential mediating roles of psychological security and aging expectations. The research conclusions are as follows: marital satisfaction positively correlates with spiritual well-being, and psychological security and aging expectations play independent chain mediating roles.

## 1. Introduction

“The key to determining whether a society is happy or not lies in the degree of happiness of the elderly”. By 2020, the proportion of the elderly population aged 60 years and above in China will exceed 17 percent, with a total population of 248 million; by 2050, the total elderly population will exceed 400 million, and the level of aging in China will enter the “fast lane” of growth [1].

### 1.1. Marital Satisfaction and Spiritual Well-Being

Spiritual well-being is an essential component of positive aging in the elderly [2]. It refers to the positive spiritual experiences such as tranquility, peace, a sense of meaning and value, hope, and strength that an individual experiences when dealing with the relationship between the self and other people, society, and the environment through self-awareness and realization to achieve the forgetfulness of the self [3]. The physical and spiritual health status of older adults is closely related to spiritual well-being [4], especially in the context of coping with the physical challenges, psychological level of adaptation, and changes in social relationships triggered by aging. During their life cycle, older people are often plagued by chronic diseases and aging problems, such as chronic pain and cognitive decline [5,6], which are closely related to depressed mood, feelings of helplessness, and a decrease in life satisfaction [7,8]. Among them, the decline in life satisfaction may stem from older people losing control over their lives and a sense of uncertainty about the future [9]. Studies have shown that spiritual well-being plays a buffering role in this process, helping older people better cope with physical and mental health problems, enhance psychological resilience, and effectively deal with the multiple challenges of aging [10,11]. In addition to age, health status, and social environment, which can affect the spiritual well-being of older adults, an individual’s internal psychological quality is also a major factor affecting the spiritual well-being of older adults [12]. Social-emotional choice theory suggests that older adults become more concerned about their emotional satisfaction as they age and are more willing to spend time with family and friends to gain emotional satisfaction. It involves an individual’s emotional experience and choices in social relationships, which suggests that people will emotionally choose relationships that provide satisfaction and support [13]. Marital satisfaction can be considered one of the critical factors influencing relationship choice in social-emotional choice theory, which refers to the level of satisfaction or happiness felt by couples in their marital relationship and is a subjective feedback and evaluation [14]. It reflects the partnership’s positive experiences and emotional connections, and individuals are more likely to choose partnerships that meet their emotional needs, provide support, and bring fulfillment, thereby increasing overall spiritual well-being [15]. Previous studies have found some gender and age differences in spiritual well-being, but no stable and consistent pattern exists across groups [16,17,18]. While most of the studies on marital satisfaction of the elderly have been conducted on attachment patterns and less on the spiritual well-being of the elderly, several studies have shown that the quality of marital relationships is closely related to the well-being of older people [19], especially in terms of mental health and emotional support [20,21]. The relationship between marital satisfaction and the spiritual well-being of older adults is mainly reflected through mechanisms such as emotional support, meeting each other’s emotional needs, reducing feelings of loneliness, and promoting positive social interactions [22]. Specifically, older adults with higher marital satisfaction receive more emotional support and a sense of belonging in their partner relationships, and these factors can significantly enhance their spiritual well-being [23]. In addition, studies have shown that marital satisfaction can further promote the spiritual well-being of older adults by enhancing the quality of life and reducing psychological stress [24]. Therefore, this study intends to examine the relationship between marital satisfaction and spiritual well-being among older adults and proposes Hypothesis 1: Marital satisfaction among older adults significantly predicts their spiritual well-being.

### 1.2. Psychological Security as a Mediator

The couple dynamic model is used to understand how interactions between spouses or partners affect the marriage’s quality and the individual’s psychological state. It focuses on factors such as emotional communication, conflict resolution, attachment, and mutual support between spouses [25], emphasizing how interactions in the relationship shape each other’s mental health and well-being [26]. The internal working model is essential in the dynamic couple relationship model. Bowlby first proposed it [27], and it was later expanded and explained by Bartholomew and Horowitz. It explains how individuals develop cognitive frameworks about themselves and others based on early attachment relationships [28]. Psychological safety is a sense of anticipation of possible physical or spiritual danger and a feeling of power or powerlessness in the face of personal treatment, which is dominated by a sense of certainty and controllability [29]. The internal working model formed by individuals in early attachment relationships affects their emotional responses and sense of trust in intimate relationships in adulthood. Securely attached couples are more likely to feel psychologically secure through stable, trusting relationships. This sense of security can reduce anxiety and loneliness, enhancing spiritual well-being [30]. Studies have shown that psychological safety helps reduce life stress and promotes spiritual well-being by increasing self-efficacy and coping skills [31,32]. Older adults also need to feel that they can express their emotions within their family and social circles without fear of being criticized or dismissed, and receiving emotional support and understanding from family and friends is essential for a sense of psychological security [33]. Given that high marital satisfaction contributes to an individual’s ability to socially interact and express emotions [34], it improves older adults’ sense of psychological security. Thus, marital satisfaction, through trust, emotional support, and a sense of dependence on the internal working model, not only enhances psychological security but also further promotes the spiritual well-being of older adults, and this study anticipates that psychological security can be used as a psychological mechanism to explain the role of marital satisfaction on spiritual well-being in older adults. Therefore, Hypothesis 2 is proposed: Psychological security plays a mediating role in marital satisfaction and spiritual well-being.

### 1.3. Aging Expectations as Mediator

Along with the social context of active aging, expectations of old age are evolving, with more focus on health, socialization, and continued learning to make the old age stage fulfilling and meaningful. Aging expectations refer to the degree or level of expectation of healthy aging among older adults [35]. Research has shown that older adults’ aging expectations are closely related to their health-promoting behaviors, their acquisition of social interactions, and their level of self-control [36]. Marital relationships, as an affectionate bond, can impact the aging expectancy of older adults, and higher aging expectancy, in turn, significantly predicts the quality of life of older adults [37]. Older adults with high marital satisfaction perceive more excellent controllability and may have more expectations of life in old age. Moreover, an individual’s expectations and attitudes towards old age may be related to their spiritual well-being [38]. For example, older people with more positive expectations of aging tend to maintain a more positive emotional state and show greater resilience in the face of physical and psychological changes in old age [39]. Positive expectations may help to enhance emotional stability and reduce stress, thereby enhancing spiritual well-being to a certain extent. Thus, aging expectations may be an essential variable in the relationship between marital satisfaction and the spiritual well-being of older adults. The aging expectation level is a protective factor for increasing healthy aging among older adults. Therefore, this study proposes Hypothesis 3: Aging expectations are a mediating variable in the relationship between marital satisfaction and spiritual well-being.

### 1.4. The Chain-Mediating Role of Psychological Safety and Aging Expectations

Psychological security and aging expectancy may play separate and chain mediating effects in marital satisfaction and older adults’ spiritual well-being. Psychological security plays a bridging role in marriage, strengthening mutual trust and emotional ties between partners, and a good sense of psychological security promotes individuals’ level of aging expectations. At the same time, a positive marital experience may motivate individuals to view the aging stage more positively and to expect more happiness and fulfillment. This process involves several dimensions: emotion, trust, and expectations. And this matching of expectations for old age and experiences in life provides better psychological and physical health for older adults [40,41]. Based on this, this study proposes Hypothesis 4: Psychological security and aging expectations play a chain mediating role in the relationship between marital satisfaction and spiritual well-being. Through this study, it helps to understand the influencing mechanisms of the spiritual well-being of the elderly, which provides a reference for future interventions of spiritual well-being in the elderly and is also of great significance for improving the level of well-being and achieving positive aging.

## 2. Methods

### 2.1. Participants and Procedure

In this study, a convenience sampling method was adopted to select a number of community- and home-bound older adults in Sichuan Province as subjects. A questionnaire survey was conducted through semi-structured interviews, with 409 questionnaires collected. After excluding invalid questionnaires, 367 valid questionnaires were finally obtained, with an effective recovery rate of 89.73%, among which 169 (46.1%) were male older adults and 198 (53.9%) were female older adults. The average age of the participants was recorded as being 64.59 ± 3.28 years, with an age range of 60–88 years.

The investigators were professionally trained graduate students in psychology who assessed the scales using a unified guide. They distributed paper questionnaires, which took more than 50 min to complete, on a community basis and collected the questionnaires on the spot after they were answered. Quality control personnel checked the questionnaires to eliminate those with missing basic information, duplicate choices, and too many missing items.

### 2.2. Measures

#### 2.2.1. Self-Administered Basic Information Questionnaire

It was used to collect general information about the subjects such as gender, age, household registration, education level, marital status, number of children, residence, income, chronic diseases, and sleep time.

#### 2.2.2. Marital Satisfaction

Marital satisfaction among older adults was assessed using the marital satisfaction subscale of the ENRICH Marital Quality Questionnaire [42], which consists of 12 core factors. The marital satisfaction subscale consists of 10 questions that require subjects to rate from 1 (completely disagree) to 5 (completely agree), measuring satisfaction with ten aspects of marriage to determine overall satisfaction. The higher the score, the more satisfied the marriage. In this study, Cronbach’s α was 0.94.

#### 2.2.3. Spiritual Well-Being

The Spiritual Index Well-being (SIWB) [43] developed by Daaleman et al. was used. Ren Yuja et al. [44] sinicization for assessing the level of spiritual well-being of older adults in the community. The SIWB is a 12-item scale used to assess an individual’s perception of the quality of their spiritual life. The SIWB is scored using a Likert 5-point scale that requires subjects to rate from 1 (strongly agree) to 5 (strongly disagree), with a total score between 12 and 60, with the higher scores indicating a stronger sense of spiritual well-being. The higher the score, the stronger the spiritual well-being. In this study, Cronbach’s α was 0.94.

#### 2.2.4. Psychological Security

In this study, we used the Sense of Security Scale (SQ) developed by Cong Zhong et al. [29], which consists of 16 items (e.g., “I never dare to refuse a friend’s request”), and this scale contains two dimensions, namely the dimension of certainty of regulation and the dimension of interpersonal security, of which there are a total of 8 items in the dimension of certainty of regulation, and the dimension of interpersonal security also includes 8 items. The scale is rated on a 5-point scale (with scores from 1 to 5 indicating very much in line to very much out of line, respectively), which requires the calculation of two sub-dimension scores and a total scale score, with the higher the score, the higher the sense of security. In this study, Cronbach’s α was 0.93.

#### 2.2.5. Aging Expectations

The Expectations of Aging Scale (ERA-12) was compiled by Sarkisian et al. [35] in 2005 and Sinicized by Li et al. [45] to assess older adults’ expectations of aging, and the Chinese version of the scale has a Cronbach’s alpha coefficient of 0.857. The scale consists of a total of 12 items in 3 dimensions and is rated on a Likert 4-point scale from 1 (strongly agree) to 4 (strongly disagree), with a total score between 12 and 48, and the higher the score, the higher the level of expectation of aging. In this study, Cronbach’s α was 0.89.

### 2.3. Data Analysis

SPSS25.0 was utilized for the data analysis. An independent sample *t*-test and an analysis of variance were used to compare the scores of various scales for older people with different characteristics. Pearson correlation analysis was used to explore the correlation between the variables. The mediating effects were tested using Model 6 in the SPSS macro program PROCESS 4.1 prepared by Hayes. In order to consider potential confounding factors, the main analysis controlled for gender, age, education level, and household registration. Chained mediation effect analysis was conducted using the bootstrap sampling method and process, with 5000 bootstrap samples taken to estimate 95% confidence intervals for various effects; a standard method bias test was conducted using validated factor analysis. Test level α = 0.05.

## 3. Results

### 3.1. Common Method Deviation Test

Since this study was based on a questionnaire, there may be a problem of standard method bias [46]; for the possible common method bias in this study, anonymity and forward and reverse scoring were used to control it during the data collection process. A validated factor analysis was used to test for common method bias on all self-assessment items, and the results showed that the model was poorly fitted, χ2/df = 4.469, CFI = 0.71, GFI = 0.50, AGFI = 0.45, NFI = 0.65, RMSEA = 0.10, so there is no serious common methodological bias.

### 3.2. Comparison of General Demographic Characteristics with Scores on Various Variables

Older people with a monthly income of more than 4000 yuan scored higher on marital satisfaction than other older people. Older people whose children visited them at least once a week scored higher on marital satisfaction, psychological security, aging expectation, and spiritual well-being than other older people. Older people who exercised three times a week or more scored higher on marital satisfaction, psychological security, aging expectations, and well-being than other older people, as shown in Table 1.

### 3.3. Correlation Analysis

Correlation analyses were conducted on the main study variables; the results are shown in Table 2. Marital satisfaction is significantly and positively correlated with psychological security, aging expectations, and spiritual well-being among older adults. Psychological security was significantly and positively associated with aging expectations and spiritual well-being, while aging expectations were significantly and positively associated with psychological security.

### 3.4. Regression Analysis of Variables

After controlling for gender, age, educational level, and household registration type, the results of the regression analysis (Table 3) show that marital satisfaction significantly and positively predicts the spiritual well-being of the elderly (*β* = 1.07, *t* = 29.53, *p* < 0.001). After incorporating psychological security and aging expectations into the regression equation, marital satisfaction significantly and positively predicts psychological security (*β* = 0.91, *t* = 15.95, *p* < 0.001) and aging expectations (*β* = 0.13, *t* = 3.46, *p* < 0.001). Psychological security significantly and positively predicts aging expectations (*β* = 0.18, *t* = 6.52, *p* < 0.001) and significantly and positively predicts the spiritual well-being of the elderly (*β* = 0.25, *t* = 7.96, *p* < 0.001). Aging expectations significantly and positively predict the spiritual well-being of the elderly (*β* = 0.15, *t* = 2.71, *p* < 0.01). At this point, the predictive effect of marital satisfaction on the spiritual well-being of the elderly is still significant (*β* = 0.79, *t* = 18.54, *p* < 0.001).

### 3.5. Chain-Mediating Effect of Psychological Security and Aging Expectations

**Figure 1:**
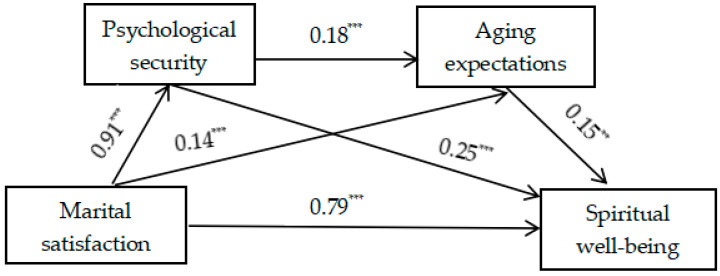
The chain-mediating role of psychological security and aging expectations in the relationship between marital satisfaction and spiritual well-being. Note. ** *p* < 0.01, *** *p* < 0.001.

The analysis of the mediating effect shows that psychological security and aging expectations have a significant mediating effect on the relationship between marital satisfaction and the spiritual well-being of older people, with a mediating effect of 0.274. The mediating effect is produced through three mediating paths. The indirect effect for path 1, ”marital satisfaction → psychological security → spiritual well-being”, was estimated to be 0.228, with a 95% confidence interval [0.143, 0.325]. The indirect effect for path 2, ”marital satisfaction → aging expectations → spiritual well-being”, was found to be 0.021, with a confidence interval of [0.004, 0.049]. The indirect effect for path 3, “marital satisfaction → psychological security → aging expectations → spiritual well-being”, was determined to be 0.025 (95% CI: [0.007, 0.045]). See Table 4. The mediating model of the relationship between the four is shown in Figure 1. The values in Figure 1 are non-standardized.

In summary, the mechanism by which marital satisfaction affects the spiritual well-being of the elderly includes direct and indirect effects. The indirect effect consists of three paths, and the 95% confidence intervals of the indirect effects of the three paths do not contain the value of 0, indicating that all three indirect effects have reached a significant level.

## 4. Discussion

This study found that older adults with a monthly income of more than 4000 yuan scored significantly higher regarding marital satisfaction than those in other income brackets. This suggests that higher socioeconomic status may provide more protection for the quality of marriage among the elderly. For example, better financial conditions can reduce financial pressures in the family, promote emotional communication between husband and wife, and thus enhance marital satisfaction [47]. This result is consistent with previous research on the positive impact of socioeconomic status on spiritual well-being [48]. Therefore, improving the financial support of the elderly may help improve the quality of their marriage and spiritual well-being. Older adults visited at least once a week by their children scored higher than other older adults in terms of marital satisfaction, psychological security, aging expectations, and spiritual well-being. According to the theory of social-emotional choice, older adults tend to optimize and maintain high-quality emotional relationships for psychological comfort and satisfaction [49]. Regular family visits and interactions provide emotional support and satisfy older adults’ attachment needs for family love, enhancing their sense of psychological security and positive expectations for the future [50]. This finding suggests that the care and companionship of children play an essential role in enhancing the spiritual well-being of older adults, further emphasizing the importance of family connections and emotional support in the well-being of older adults [51]. The study also found that older adults who exercised three or more times a week scored significantly higher in regard to marital satisfaction, psychological security, aging expectations, and spiritual well-being than those who exercised less. This suggests that an active lifestyle is closely linked to older adults’ psychological and spiritual well-being. Not only does regular exercise help to keep the body healthy, but it may also promote mental health by increasing psychological security and self-efficacy [10,52]. The results show that marital satisfaction can directly and positively predict the spiritual well-being of the elderly, which verifies Hypothesis 1. This is consistent with the results of domestic and foreign studies [53]. The elderly with high marital satisfaction tend to have higher well-being because, compared with middle-aged couples, elderly couples have better emotional control in their marital relationships. This is because their construction helps to improve the growth environment for happiness [13]. This study also found that marital satisfaction has a direct positive effect on older adults’ spiritual well-being and indirectly affects spiritual well-being through the mediating effects of psychological security and aging expectations. Among them are three paths of mediating effects: the independent mediating effect of psychological security, the independent mediating effect of aging expectations, and the chain mediating effect of psychological security and aging expectations.

### 4.1. The Mediating Role of Psychological Security

This study found that psychological security has a separate mediating effect between marital satisfaction and the spiritual well-being of the elderly, which verifies Hypothesis 2. According to Bowlby’s attachment theory, the attachment patterns formed by individuals in early life affect how they establish relationships with others in adulthood. Individuals with positive internal working models tend to feel more support and trust in intimate relationships, enhancing psychological security [54]. The elderly with higher marital satisfaction will use fewer strategies of blaming others and silence when negative events occur between couples [55]. Previous research has also found that individuals who are satisfied with their marriage are less likely to blame the other party during negative events [56]. On the contrary, they are more likely to consider their partners and have more altruistic behaviors [57]. This shows that higher marital satisfaction can provide or make older people feel more psychological security, which in turn leads to higher levels of spiritual well-being. When couples establish psychological security, they are more likely to share emotions, communicate, and support each other in their marriage, which helps to enhance their marital satisfaction. As they get older, older people become more dependent on their marital relationships, which is particularly important for their spiritual well-being. This suggests the importance of maintaining a healthy marital relationship for older people. For example, emotional support between spouses can help older people improve their marital relationships. Regular exercise can also improve older people’s spiritual well-being and enhance their positive psychological qualities [58].

### 4.2. The Mediating Role of Aging Expectations

This study also found that aging expectations mediate between marital satisfaction and older adults’ spiritual well-being, which verifies Hypothesis 3. According to the theory of internal working models, attachment relationships formed early in life affect an individual’s current intimate relationships and expectations for the future [59]. Individuals with positive internal working models tend to have a positive attitude toward the future, showing greater resilience when facing aging [60]. Previous studies have shown that marital status particularly impacts aging expectations. The aging expectations of older people with spouses are higher than those of older people without spouses, possibly due to family support and companionship [61]. The care from family members, especially spouses, can provide psychological support for the elderly and prevent the formation of negative emotions [62]. In addition, some studies have found that the support of family members and friends significantly improves the subjective well-being and life satisfaction of the elderly, among which family support plays a more significant role, especially in terms of emotional support [63]. In other words, the family functions as an essential entry point for intervention [64], which can enhance the well-being of older adults by encouraging family members to provide support and understanding, promote positive family interactions, and encourage positive aging expectations.

### 4.3. The Chained Mediating Effects of Psychological Security and Aging Expectations

This study also found that psychological safety and aging expectations have a chain mediating effect between marital satisfaction and the spiritual well-being of the elderly, verifying Hypothesis 4. This shows that different levels of psychological security affect individuals’ expectations of aging. The higher the marital satisfaction, the higher the individual’s psychological security and thus, the more potent the ability to express emotions. This also enhances the security experience of the elderly. The elderly with a high ability to express emotions can communicate their wishes to family members and obtain feedback from them, thus gaining a sense of security [65,66]. They are more likely to provide themselves with more positive psychological resources [67], which enhances their psychological security and expectations of aging and ultimately positively affects the spiritual well-being of the elderly. The chain mediation between psychological safety and aging expectations shows that the mechanism of the impact of marital satisfaction on the spiritual well-being of the elderly is not a single one; instead, it works through psychological safety and the positive aging process of the elderly to further influence the cognitive processes of the elderly. This suggests that as people age, they can help couples plan their lives in their later years together, set goals and make plans together, and promote the tendency of the elderly to have a higher positive emotional attitude towards the future.

### 4.4. Limitations and Implications

This study has the following limitations: (1) This research is a cross-sectional survey, and no causal relationship can be drawn. In the future, longitudinal or intervention studies on the spiritual well-being level of the elderly can be conducted to explore its development process. (2) The selection of the research subjects in this study was the elderly in Sichuan Province, and there may be a problem of insufficient sample representativeness. Future studies can further expand the scope of the study to improve the credibility of the conclusions. (3) While a positive effect of certain mediating variables on marital satisfaction was hypothesized in this study, the discussion mentions the possibility of an inverse relationship whereby the mediating variables may, in turn, affect marital satisfaction. This suggests another interpretation of model directionality. Therefore, the model structure of this study may not have fully captured the relationship between the variables, and future research should consider further validation of this reverse path to ensure the robustness of the model.

Combining the findings of this study, marital satisfaction, psychological security, and aging expectations all significantly impacted older adults’ spiritual well-being. Psychological security and aging expectations functioned as independent mediating variables and also explained how marital quality affects spiritual well-being through psychological mechanisms. These results suggest that older adults’ spiritual well-being is enhanced by their emotional support in social relationships and positive expectations for the future. This comprehensive analysis supports the research hypothesis that marital quality affects older adults’ spiritual well-being through complex psychological and cognitive processes.

The results of this study not only enrich our understanding of older adults’ spiritual well-being at the theoretical level but also provide essential insights for interventions in practice. First, the significant effect of marital satisfaction on spiritual well-being suggests that improving marriage quality can be an essential direction for intervening in older adults’ mental health. Therefore, marriage counseling and emotional support programs may be essential means for older couples to improve their spiritual well-being. In addition, the mediating effect of psychological security further suggests that interventions targeting older adults’ psychological security, such as mental health counseling or emotional support services, can effectively promote their spiritual well-being. Finally, the positive effect of aging expectations suggests that policymakers and communities can improve the quality of life of older adults by promoting the concept of active aging. These findings provide a practical basis for developing more targeted mental health policies for older adults.

## 5. Conclusions

This study reveals that marital satisfaction is significantly and positively correlated with the spiritual well-being of the elderly. Psychological security and aging expectations have a partial chain mediating effect on marital satisfaction and the spiritual well-being of the elderly; that is, marital satisfaction can directly affect the spiritual well-being of the elderly and can also improve spiritual well-being by increasing psychological security or enhancing the aging expectations of the elderly. This study helps to raise social awareness of the elderly to meet their needs better, promote active aging, and enable the elderly to lead happier and more meaningful lives in their later years.

## Figures and Tables

**Table 1:** Comparison of general demographic characteristics with scores on various variables.

Group		N	Marital Satisfaction	Psychological Security	Aging Expectations	Spiritual Well-Being
Gender	male	169	27.85 ± 12.50	46.99 ± 17.36	22.35 ± 8.65	33.32 ± 15.54
	female	198	26.03 ± 12.09	46.40 ± 17.28	21.20 ± 7.67	32.15 ± 15.67
	*t*		1.42	0.322	1.352	0.718
	*p*		0.156	0.748	0.177	0.473
Household registration	urban	224	27.00 ± 12.07	47.39 ± 17.98	21.91 ± 8.49	33.39 ± 16.10
	rural	143	26.66 ± 12.68	45.55 ± 16.16	21.44 ± 7.60	31.59 ± 14.76
	*t*		0.241	0.992	0.539	1.079
	*p*		0.802	0.322	0.590	0.281
Marital status	spouse	362	26.83 ± 12.29	46.59 ± 17.31	21.74 ± 8.18	32.72 ± 15.62
	spouseless	5	29.40 ± 13.87	52.60 ± 17.05	20.60 ± 6.11	30.20 ± 15.52
	*t*		−0.463	−0.771	0.311	0.358
	*p*		0.643	0.441	0.756	0.720
Number of chronic diseases	not	100	26.60 ± 11.85	48.13 ± 17.44	21.96 ± 7.81	33.10 ± 15.73
	1 type	118	26.55 ± 12.23	45.47 ± 17.32	21.97 ± 8.55	32.20 ± 15.60
	2 or more types	149	27.30 ± 12.70	46.65 ± 17.22	21.38 ± 8.08	32.80 ± 15.61
	*F*		0.152	0.641	0.233	0.095
	*p*		0.859	0.527	0.792	0.910
Number of visits by children	once a week ①	234	28.15 ± 12.54	48.47 ± 17.98	22.41 ± 8.54	34.85 ± 16.00
	once a fortnight ②	70	26.89 ± 12.39	45.26 ± 15.74	22.37 ± 7.99	32.66 ± 14.75
	once a month ③	58	21.17 ± 9.49	40.90 ± 15.64	18.00 ± 5.37	24.26 ± 12.31
	once a year ④	5	32.80 ± 11.71	49.40 ± 8.23	23.80 ± 8.35	29.60 ± 10.78
	*F*		5.580	3.255	5.016	7.600
	*p*		<0.001	0.022	0.002	< 0.001
	compare pairwise (*p* < 0.05)		① > ②, ② > ③, ③ > ④	① > ②	① > ②, ② > ③	① > ②, ② > ③
Frequency of physical exercise	never ①	126	22.06 ± 10.07	40.82 ± 13.93	19.92 ± 5.74	26.44 ± 11.50
	1–2 times a week ②	120	26.46 ± 12.39	46.07 ± 16.14	21.24 ± 8.13	31.88 ± 15.07
	3+ times/week ③	121	32.28 ± 12.21	53.37 ± 19.25	2409 ± 9.66	40.00 ± 16.84
	*F*		24.155	17.876	8.761	26.913
	*p*		<0.001	<0.001	<0.001	<0.001
	compare pairwise (*p* < 0.05)		③ > ② > ①	③ > ② > ①	③ > ② > ①	③ > ② > ①
Personal monthly income	<1000 ①	5	35.60 ± 7.80	54.20 ± 7.89	25.60 ± 5.13	40.60 ± 5.73
	1000–1999 ②	57	26.75 ± 11.83	46.00 ± 17.03	21.40 ± 8.11	32.58 ± 15.12
	2000–2999 ③	262	25.87 ± 12.24	46.19 ± 17.19	21.85 ± 8.27	31.76 ± 15.56
	3000–3999 ④	36	30.86 ± 12.88	48.72 ± 20.32	20.36 ± 8.28	37.17 ± 17.39
	>4000 ⑤	7	38.14 ± 6.64	54.29 ± 8.88	24.14 ± 3.38	39.71 ± 11.23
	*F*		3.578	0.772	0.724	1.665
	*p*		0.007	0.544	0.576	0.157
	compare pairwise (*p* < 0.05)		⑤ > ①, ⑤ > ③, ④ > ③			
Education level	primary and below	111	26.13 ± 12.12	46.25 ± 16.93	21.48 ± 7.93	31.86 ± 15.07
	junior	254	27.13 ± 12.38	46.76 ± 17.51	21.80 ± 8.27	33.07 ± 15.87
	high school	2	35.00 ± 14.14	59.00 ± 9.90	26.50 ± 4.95	29.50 ± 13.43
	*F*		0.694	0.543	0.404	0.277
	*p*		0.500	0.582	0.668	0.758

Note. N = 367.

**Table 2:** Correlations among the main study variables.

	1	2	3	4
1. Marital satisfaction				
2. Psychological security	0.64 **			
3. Aging expectations	0.45 **	0.51 **		
4. Spiritual well-being	0.84 **	0.72 **	0.51 **	

Note. N = 367, ** *p* < 0.01 two-tailed.

**Table 3:** Relationships of variables in the chain mediation model.

Variables	Spiritual Well-Being		Psychological Security		Aging Expectations		Spiritual Well-Being	
	*β*	*t*	*β*	*t*	*β*	*t*	*β*	*t*
Gender	0.81	0.91	1.10	0.78	−0.80	−1.09	0.63	0.79
Age Education level	0.03 −0.99	0.25 −0.95	−0.18 −1.04	−0.08 −0.63	0.01 0.13	0.09 0.15	0.03 −0.73	0.23 −0.78
Household registration	−1.88	−1.89	−1.97	−1.26	−0.05	−0.06	−1.31	−1.48
Marital satisfaction	1.07	29.53 ***	0.91	15.95 ***	0.14	3.46 ***	0.79	18.54 ***
Psychological security					0.18	6.52 ***	0.25	7.96 ***
Aging expectations							0.15	2.71 **
*R* ^2^	0.71		0.42		0.29		0.77	
*F*	176.27 ***		51.46 ***		25.15 ***		171.80 ***	

Note. N = 367, ** *p* < 0.01, *** *p* < 0.001.

**Table 4:** Psychological security and aging expectations in the mediation effects analysis.

	Effect	Boot SE	Boot LLCI	Boot ULCI	Percentage of Total Effect (%)
Model pathways					
Indirect effect	0.274	0.047	0.187	0.371	25.84%
Indirect effect 1	0.228	0.046	0.143	0.325	21.50%
Indirect effect 2	0.021	0.011	0.004	0.049	1.98%
Indirect effect 3	0.025	0.010	0.007	0.045	2.36%

Note. N = 367. LL = low limit; CI = confidence interval; UL = upper limit. Indirect effect 1: Marital satisfaction → psychological security → spiritual well-being; indirect effect 2: Marital satisfaction → aging expectations → spiritual well-being; indirect effect 3: Marital satisfaction → psychological security → aging expectations → spiritual well-being.

## Data Availability

The datasets used and analyzed in the current study are available from the corresponding author upon reasonable request.
